# Platelet-leukocyte aggregates – a predictor for acute kidney injury after cardiac surgery

**DOI:** 10.1080/0886022X.2021.1948864

**Published:** 2021-07-15

**Authors:** Shenghan Yang, Xunbei Huang, Juan Liao, Qin Li, Si Chen, Chaonan Liu, Liqin Ling, Jing Zhou

**Affiliations:** Department of Laboratory Medicine, West China Hospital, Sichuan University, Chengdu, China

**Keywords:** Acute kidney injury, platelets-leukocyte aggregates, cardiac surgery

## Abstract

**Background:**

Acute kidney injury (AKI) is one of the most common complications after cardiac surgery. However, effective biomarker used for early diagnosis of AKI has not been identified. Platelet-leukocyte aggregates (PLAs) participate in inflammation and coagulation, leading to vascular lesions and tissue destruction. We designed a prospective study to assess whether PLAs can serve as a good biomarker for early diagnosis of AKI after cardiac surgery.

**Methods:**

Patients with rheumatic heart disease scheduled to undergo valve replacement surgery were enrolled. Blood samples were collected at five timepoints as follows: (a) At baseline. (b) At the end of extracorporeal circulation. (c) Arrival at intensive care unit (ICU). (d) Four-hours after the admission to ICU. (e) Twenty hours after the admission to ICU. After collection, the samples were immediately used for PLAs measurement by flow cytometry.

**Results:**

A total of 244 patients were registered, and 15 of them were diagnosed with AKI according to the serum creatinine of KDIGO guidelines. The PLAs levels in AKI group were significantly increased 20 h after surgery (two-way repeated measure analysis of variance, *p* < 0.01) compared with that at baseline. Patients whose preoperative PLAs were higher than 6.8% showed increased risk of developing AKI (multivariate logistic regression; *p* = 0.01; adjusted odds ratio, 1.05; 95% confidence interval, 1.01–1.09).

**Conclusion:**

PLAs is an independent risk factor for AKI after valve replacement among patients with rheumatic heart disease.

## Introduction

Acute kidney failure (AKI) is a worldwide public health concern, which put a heavy economic burden to the health care system. AKI frequently occurs in hospitalized patients and critically ill patients with an incidence ranging from 2 to 66%, relevant to the formation of chronic kidney disease and other adverse outcomes and associated with a higher risk for mortality and longer hospital stay [1,2]. About 10 ∼ 50% of patients with kidney failure require renal replacement therapy [3,4]. Even an episode of stage 1 AKI (increase of serum creatinine level < two-fold from baseline) may affects for more than 10 years [5].

Until today, apart from supportive care, limited effective therapy is available for AKI. Early identification and intervention are important to prevent the deterioration of AKI. However, a nationwide cross-sectional survey reported that nonrecognition or nondocument rate of AKI was up to 74.2% in different provinces in China [6]. Patients often presented with atypical clinical manifestations and accompanied with underlying diseases, which made the timely diagnosis of AKI difficult. Cardiovascular surgery-related acute kidney injury (CSA-AKI) is one of these complex disorders. More than 2 50 000 patients undergo cardiovascular surgery annually in China, with an incidence rate of CSA-AKI ranging from 5 to 42% [6–9]. Urine output and serum creatinine are used as traditional indicators to make a definite diagnosis for AKI, but cannot be used for early diagnosis. Novel biomarker, such as neutrophil gelatinase-associated lipocalin (NGAL), was shown to be useful for early identification of AKI. NGAL, however, also increased in other conditions and thus is lack of reasonable specificity [10,11] and cannot accurately recognize high-risk patients among septic ICU patients. New diagnostic biomarkers are imperative to screen high-risk patients and improve their renal outcome.

Platelet-leukocyte aggregates (PLAs), formed by combination of platelet with leukocytes, participate in inflammation and coagulation *via* destroying vascular endothelium and result in tissue destruction and organs dysfunction, especially those with abundant vascularity like kidney and lung. Studies have confirmed that PLAs are associated with adverse cardiac and cerebrovascular events after valve replacement surgery [12]. Based on this pathophysiology, our study aims to explore the relationship of the level of PLAs and the associated risk of developing acute kidney injury after cardiac surgery.

## Methods

### Patients

Our cohort enrolled adult patients (18–65 years old) with rheumatic valve disease who underwent surgical intervention at West China Hospital, Sichuan University between November 1, 2011, and September 30, 2012. Patients with following conditions were excluded: (a) known organ dysfunction, including adult respiratory distress syndrome (ARDS), kidney failure or New York Heart Association (NYHA) class IV heart failure, (b) preoperative pulmonary diseases including chronic obstructive pulmonary disease, pneumonia, and pulmonary hypertension, (c) confirmed systemic inflammatory response syndrome (SIRS) . (d) history of any heart surgery , (e) required a second operation because of hemorrhage or other adverse event, and (f) patients who have been or are participating in other clinical studies. Detailed definitions of above diseases are provided in Supplemental Information 1.

Every patient signed written informed consent. The study protocol was approved by the Ethics Committee of Sichuan University (approval number: 2011-133) and conducted according to the Declaration of Helsinki. This study was registered in the Chinese Clinical Trial Registry (ChiCTR-OCH-12001922).

### Outcome definition

Primary outcome was the development of AKI, defined based on KDIGO guidelines [13]: 0.3 mg/dL increase or ≥50% increase in serum creatinine level from baseline preoperative level to postoperative level at any time during the hospital stay and that could not be explained by fluid resuscitation, overload, or dialysis.

### Laboratory testing

Arterial blood samples were collected into an ethylenediaminetetraacetic acid -pretreated vacutainer (BD Basel, Switzerland) after anesthesia induction. The next four specified timepoints for blood draw were: end of extracorporeal circulation, 0 h, 4 h, and 20 h in the intensive care unit (ICU). Samples were incubated with CD62L-PE (BD Basel), CD41a-FITC (BD Basel), and DRAQ5 (Biotium, Hayward, CA). An IgG1-FITC/PE antibody (BD Basel) was used as an isotype control. After immunolabelling, cell nuclei were stained by DRAQ5, and PLAs were measured by CD62L/CD41a dot plots by flow cytometry (Beckman Coulter FC500 Flow Cytometer, Beckman, UK). In addition, the samples were stained with FACSAria and PLAs could be observed and confirmed under a confocal fluorescence microscope. Details of PLAs measurements are provided in Supplemental Information 2. All samples for flow cytometry, leukocyte counts, and neutrophil counts were processed within 2 h after collection.

We collected heparinized arterial blood samples at the same time as previously mentioned and separated the plasma after centrifugation at 4 °C for 15 min at 1000 g. Tumor necrosis factor - alpha (TNF-α) and interleukin- 8 (IL-8) were measured with commercial enzyme-linked immunosorbent assay kits (High Sensitivity Kit, R&D Systems, Minneapolis, MN). Plasma samples was stored at −80 °C and assays for TNF-α and IL-8 were carried after sample collection were completed for all the participants.

### Statistical analyses

Data are presented as percentages for categorical data, mean ± standard deviation for normally distributed data, median with lower and upper quartiles for non-normal distributed data. Two-way repeated measure analysis of variance with the Bonferroni *post hoc* test was used to compare changes of each variable over time. Univariate and multivariate logistic regression were used to analyze risk factors associated with the outcome. Three factors with a significance level of 0.1 in univariate logistic regression were further enrolled in the multivariate logistic regression including sex, smoking and hypertension (Supplemental Information 3). Other candidate variables in multivariable logistic regression were chosen from highly suspected related variables in clinic and previous researches [12] including age, body mass index (BMI), NYHA classification, diabetes, atrial fibrillation, digoxin, type of valvular disease, and cardiopulmonary bypass (CPB) time. Receiver operating characteristic (ROC) curve and Youden’s index were used to determine the best cutoff value for predicting acute kidney injury. Patients were classified into two groups through the cutoff value.

A two-sided *p* value < 0.05 was considered statistically significant. SPSS (version 19. 0, IBM, Chicago, IL) was used for statistical analysis.

## Results

### Demographic characteristics

A total of 244 patients were enrolled in this study with a median age of 47 years including 78 males (32%) and 166 females (68%). A total of 15 patients were diagnosed as AKI. There was no significant difference between the AKI group and the non-AKI group in age, sex, BMI, disease history and medication. The smoking rate of patients in the AKI group was significantly higher than that in the non-AKI group (Table 1).

**Table 1.** Preoperative characteristics of patients.

	Acute kidney injury		
	No (*n* = 229)	Yes (*n* = 15)	*p* Value
Age, years	47.27 ± 9.34	48.60 ± 7.69	0.61
Male, *n* (%)	70 (30.6%)	8 (53.3%)	0.12
Body mass index, kg/m²	22. 26 ± 2.80	22.45 ± 3.00	0.11
Smoking, *n* (%)	45 (19. 7%)	7 (46. 7%)	0.03
NYHA class			0.56
II	36 (15. 7%)	1 (6. 7%)	
III	193 (84. 3)	14 (93. 3%)	
Diabetes	4 (1. 7%)	1 (6. 7%)	0.72
Atrial fibrillation	112 (48. 9%)	6 (40%)	0.69
Hypertension	15 (6. 6%)	3 (20%)	0.16
Left atrial thrombus	31 (13. 5%)	2 (13. 3%)	1.00
Medications, *n* (%)			
Warfarin	2 (0.9%)	1 (6.7%)	0.45
Calcium antagonists	2 (0.9%)	0	1.00
β blocker	17 (7.4)	2 (13.3%)	0.74
Digoxin	25 (10.9%)	1 (6.7%)	0.93
Aspirin	11 (4.8%)	0	0.82
Anticoagulants	13 (5.7%)	1 (6.7%)	1.00
Insulin	1 (0.4%)	0	1.00
ACEI	9 (3.9%)	0	0.94
Diuretics	25 (10.9%)	1 (6.7%)	0.93
CBC before surgery			
Leukocytes, ×10^9^/L	4. 41 (3.52, 5.56)	4. 74 (3.31, 6.10)	0.99
Platelets, ×10^9^/L	130 (101.5, 161.50)	116 (85.25, 156)	0.09
Erythrocytes, ×10^12^/L	4.17 (3.86, 4.63)	4.43 (4.26, 4.62)	0.13
LVEF, %	63 (57, 68)	62 (58, 66)	0.88
EF SCORE			0.97
0 (LVEF, > 50%)	195 (89.4%)	14 (93.3%)	
1 (LVEF, 30%∼50%)	23 (10. 6%)	1 (6.7%)	
3 (LVEF, < 30%)	0	0	
Type of valvular disease, *n* (%)			0.04
Mitral valve	95 (41.9%)	3 (20%)	
Aortic valve	35 (15.4%)	6 (40%)	
Mitral and aortic valve	97 (42.7%)	6 (40%)	
CPB time, min	112 (90.5,136.5)	122 (97, 147)	0.23
Cross-clamp time, min	73 (55.0, 96. 5)	88 (65, 98)	0.43
Transfusion			
Red blood cells, u	0 (0, 1. 5)	0 (0, 1. 5)	0.72
Platelets, u	0 (0, 0)	0 (0, 0)	0.80
Fresh frozen plasma, ml	0 (0, 0)	0 (0, 0)	0.83

NYHA: New York Heart Association functional class; ACEI: angiotensin - converting enzyme inhibitor; CBC: complete blood count; LVEF: left ventricle ejection fraction; EF SCORE: European System for Cardiac Operative Risk Evaluation; CPB: cardiopulmonary bypass.

### CPB and inflammatory markers

**Figure 1.**
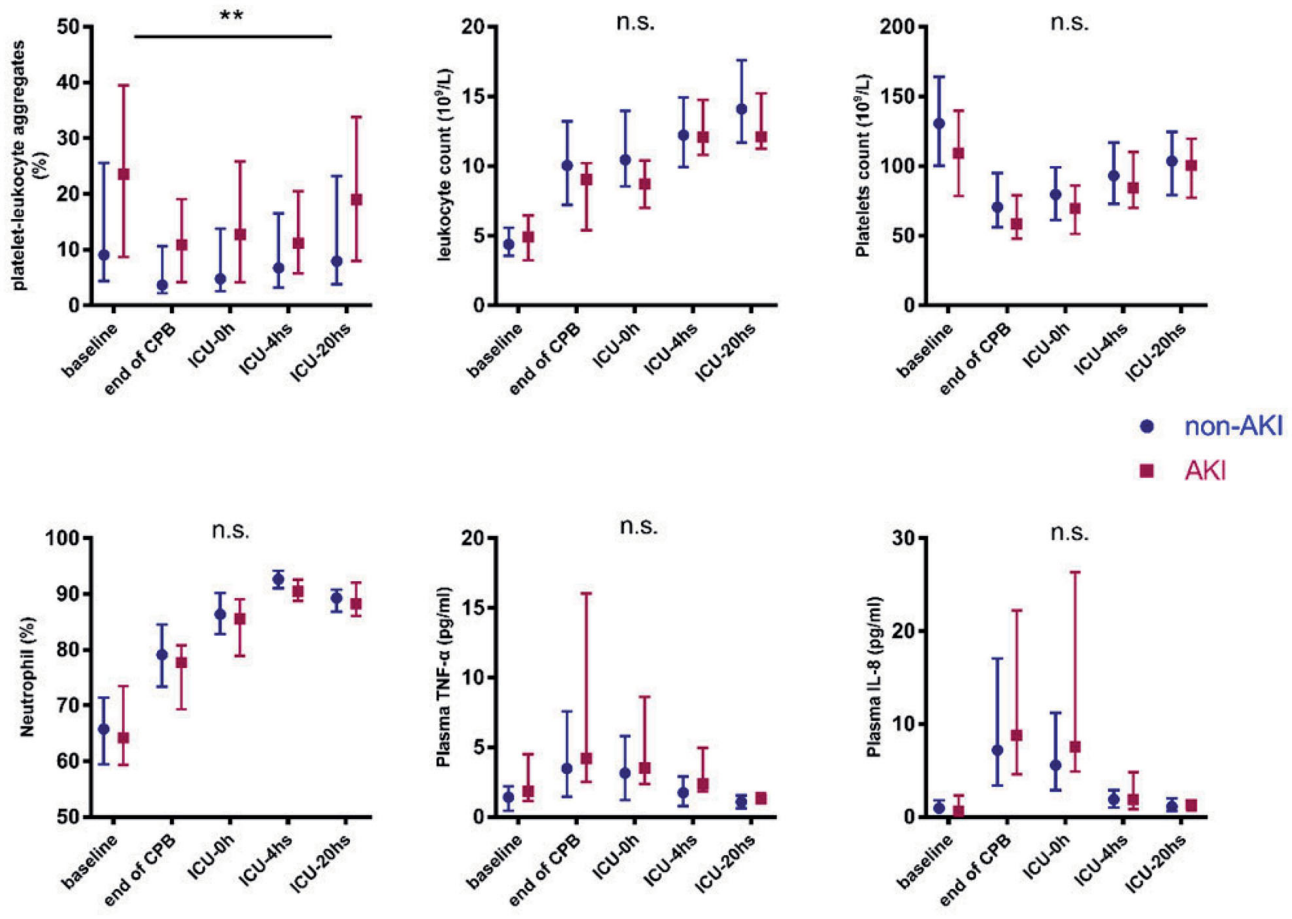
Changes of PLAs, leukocyte, platelets, neutrophils, TNF-α, and IL-8 at preoperation, end of CPB, instant, 4 h, and 20 h in intensive care unit. Squares/dots and short lines stand for median and the upper and lower quartiles. ** *p* < 0.01. n.s.: no significance. PLAs in AKI group was significantly higher than non-AKI group (*p* < 0.01, two-way repeated measure analysis of variance), while other indicators showed no significant differences between two groups.

PLAs and platelets were at the lowest level at the end of CPB and then gradually peaked at 20 h after admission to ICU. The counts of leukocytes and neutrophils presented a continuous increasing trend. TNF-α and IL-8 peaked at the end of CPB, then gradually decreased to the lowest level at 20 h in ICU, which were close to their preoperative baseline level. (Figure 1)

**Figure 2.**
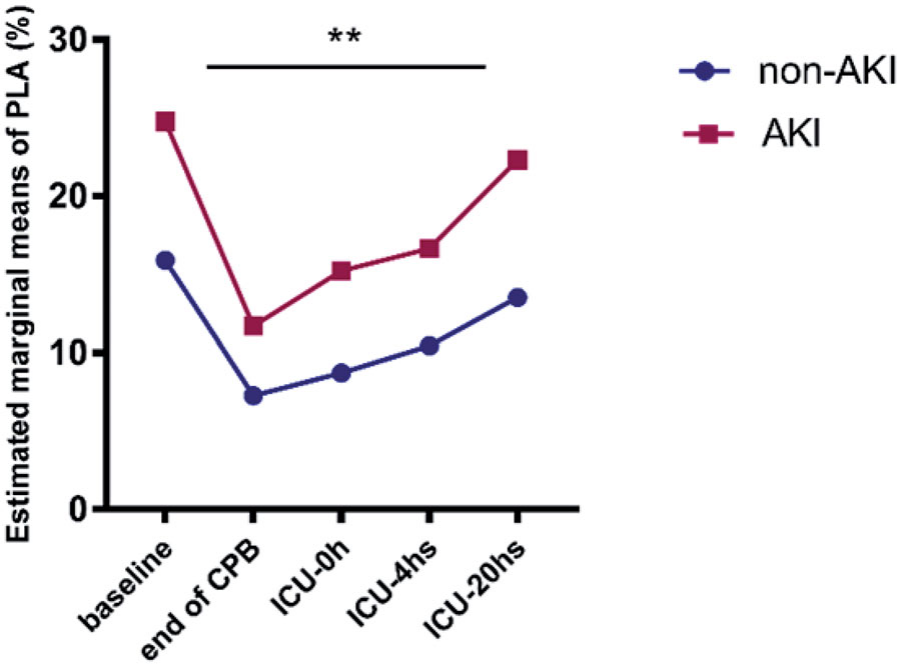
Estimated marginal means of PLAs (analyzed by two-way repeated measure ANOVA). Patients who developed AKI had significantly increased PLAs level over time (*F* = 134. 254; *p* < 0. 01). ** *p* < 0.01.

In the tests of within-subject’s effects, multivariate analysis of variance showed that timepoint interacts with the rising of PLAs level (*p* < 0.01, with Greenhouse-Geisser correction). Contrasts tests showed that the PLAs level of two groups displayed a similar increasing trend (*p* > 0.05). PLAs in AKI group was higher than that in non-AKI group from pre- to postoperative, and the tests of between-subjects effects indicated these differences were significant (*F* = 134. 254, *p* < 0.01; Figure 2). Same tests were performed to analyze the changes of white blood cells, neutrophils, platelets, TNF-α, and IL-8 between the two groups, no statistical differences were found (Supplemental Information 4).

### Preoperative parameters and PLAs

According to the results above, preoperative baseline level of PLAs was significantly higher in patients with postoperative AKI (non-AKI group vs. AKI group,15.9 ± 14.5 vs. 24.8 ± 15.3), and odds ratios of PLAs at different time points were quite close (Supplemental Information 5). Univariate logistic regression was performed to find the correlation between baseline level of PLAs and other preoperative parameters with no significant correlations between PLAs and these parameters (Supplemental Information 6).

### Preoperative baselines of PLAs and AKI

To reduce the frequency of blood draw, we simply used preoperative baseline to assess the relationship between outcome and variables. The results of univariate logistic regression showed that preoperative baseline level of PLAs was associated with development of AKI (as continuous variable, odds ratio, 1.04; 95% confidence interval [CI], 1.00–1.07; *p* = 0.03). Multivariate logistic regression analysis showed that this difference remains after adjusting by confounding factors. Each 1% of PLAs rise was significantly associated with a 5% increased risk for postoperative AKI (95% CI, 1.01–1.09; *p* = 0.01).

A ROC curve was used to identify the optimal cutoff of PLAs. As a result, a preoperative PLAs of 6.8% was determined to be the best cutoff value with sensitivity, specificity, positive predictive value, and negative predictive value of 93.3%, 40.2%, 9.3%, and 98.9%, respectively. The area under the curve (AUC) was 0.69 (95% CI, 0.59–0.81; Supplemental Information 7). The risk for AKI in patients whose preoperative PLAs > 6.8% was 18.35 times higher than that of other patients (95% CI, 1.90–177.03; *p* = 0.01; Table 2). No association was found between other preoperative indexes and AKI (Supplemental Information 8).

**Table 2.** Logistic regression analysis.

	Unadjusted		Adjusted^a^	
	OR (95% CI)	*p* Value	OR (95% CI)	*p* Value
PLAs (as continuous variable)	1.04 (1.00–1.07)	0.03	1.05 (1.01–1.09)	0.01
PLAs (as categorical variables^b^)	9. 93(1.28–76.77)	0.03	18.35 (1.90–177.03)	0.01

^a^Adjusted by sex, age, BMI, smoking history, NYHA classification, hypertension, diabetes, atrial fibrillation, digoxin, type of valvular disease, and CPB time.

^b^Categorized by PLAs = 6.8% (calculated by ROC curve).

## Discussion

In our study, PLAs were proved to be a good reflection to the long-term status of inflammation caused by underlying disease and could be helpful in early diagnosis of AKI after a valve replacement surgery. PLAs were consistently elevated in patients with postoperative AKI after a valve replacement. We demonstrated for the first time that increased level of preoperative PLAs is an independent risk factor for AKI after valve replacement among patients with rheumatic heart disease. Preoperative PLAs > 6.8% is associated with the risk for AKI by 18-fold.

Research about CSA-AKI has made great progress with the development of the emerging disciplines in recent years. Novel biomarkers, such as Kidney injury molecule 1 (KIM-1), urinary NGAL, a plasma serum cystatin C (CysC), and hepcidin regulatory protein (HJV) have been discovered in succession and brought the potentiality of early diagnosis of postoperative AKI [14–16], but some practical clinical application still suffer from the narrow applicable scope of diseases and lack of a broad international consensus. For example, NGAL is present in several types of cells. Increased serum NGAL is not only detected in patients with renal injury, but also in some malignancies [11] and serious infections [17–19]. Furthermore, regular detection for these novel biomarkers is not available in many hospitals in China. It is of great significance to search for sensitive biomarkers of AKI.

Some studies have observed increased circulating PLAs in many vascular and inflammation diseases including atherosclerosis [20], unstable angina [21], stroke [22], and inflammatory bowel disease [23]. Similar cell-cell interactions also present in our research among patients who experience a renal damage after cardiac vascular surgery. Our data shows that the abnormality of PLAs display a relatively broad time window, and approximate odds ratios present in measurements in different time. The median level of preoperative PLAs for the AKI and non-AKI group were 23.5% and 9.1%, respectively, which were both significantly higher than that in previously reported healthy population (3.6%) [22]. The abnormal accumulation of PLAs already appeared before surgery; thus, we can speculate that PLAs may be more sensitive to a preinflammatory state than other parameters and have the potential to serve as a good biomarker.

There were several characteristic change curves of other inflammatory markers like TNF-α and IL-8, but we did not see a significant correlation with the outcome. Besides, results of TNF-α and IL-8 in other clinical studies were controversial [24–27], for which different biochemical mechanisms between PLAs and other biomarkers might account. The increase of baseline level of PLAs in patients with AKI was a reflection to the long-term status of inflammation caused by underlying disease. But TNF and IL-8 show a short half-life *in vivo*, mirroring immediate inflammatory events [28,29]. The changing trends of TNF-α and IL-8 that we observed in our study may be caused by operational procedures such as cardiopulmonary bypass. Also, the prospective study design of our study allows us to better control the confounding factor compared to other retrospective studies.

Valve diseases caused by rheumatism have an intricate inflammatory pathogenesis [30]. The genesis and development of pathological changes in cardiovascular system involve a series of cells and cytokines. Leukocytes, especially neutrophils and monocytes, can produce an abundant amount of oxygen free radicals, release elastase and myeloperoxidase during polarization, adhesion, and infiltration, leading to vascular and tissue damage. Platelets have also been confirmed as a principal member participating in endothelial injury, leukocyte recruitment, microvascular thrombogenesis, and other inflammatory process [31,32]. These two kinds of cells can combine with each other and form a stable heterotypic complex through several receptor/ligand pairs, including P-selectin and CD40 ligand on platelets, P-selectin glycoprotein ligand- 1 (PSGL-1), CD40, and Mac-1 on leukocytes [33–35]. The cross-communication between platelets and leukocytes stimulates immediate release of chemokines and cytokines, creating a particular microenvironment that facilitates the recruitment of more cells to the damaged site. Also, intracellular signal pathways can be activated to induce delayed response including gene expression and protein synthesis. For example, some studies have found that P-selectin act as an inducer to the expression and transcription of IL-8 and cyclooxygenase (COX)-2 gene in monocytes [36,37]. In conclusion, PLAs is capable to facilitate the exacerbation of inflammation, worsen vascular and tissue injury [38]. Therefore, higher preoperative PLAs level in patients with preexisting inflammatory state may impose severer vascular damage.

In the meantime, we also found that after cardiopulmonary bypass, the average level of PLAs in all patients rise continuously till 20 h after operation, which implies that the excessive activation of platelets and leukocytes may be stimulated by the systemic inflammatory response after CPB. The second hit from surgical operation worsens extracellular microenvironment and thus leads to additional risk for renal dysfunction.

We also noticed that there might be a slight inconsistency in results of preoperative indexes, that is, AKI patients had higher leukocyte counts and PLAs level, while non-AKI patients had a higher platelet counts, although only differences in PLAs was statistically significant. Previous studies reported that proper or moderately elevated platelets were a protective factor for poor prognosis, although the mechanism remains unclear [39–41]. Combined with our findings that PLAs level was a potential risk factor, it is suggested that platelets might acquire capability of vascular damage by forming complex with leukocytes after activation.

Our study has limitations. First, the study inherently has a center effect and a selection bias as a single-center study. The insufficient number of patients with a single specific disease in this study may also cause bias, which also led to wide confidence intervals. Further research with larger samples besides rheumatic heart disease and multicenter verification should be planned next. Second, we did not carry out leukocyte’s classification during the detection of PLAs, which we propose to be examined in the future work to identify individual contributions of different subtypes. Besides, the AKI diagnosis only emphasized the serum creatinine, leading to underestimation of the incidence rate of AKI. Despite those limitations, our study showed that preoperative PLAs level can be used as a predictor for CSA-AKI.

## Supplementary Material

Supplemental Material
