# Supplementary material for: Platelet-leukocyte aggregates – a predictor for acute kidney injury after cardiac surgery
Source: Ren Fail. 2021 Jul 15;43(1):1155–62. doi: 10.1080/0886022X.2021.1948864 (PMC8288121; doi:10.1080/0886022X.2021.1948864)
Supplement: Supplemental Material [file IRNF_A_1948864_SM6784.pdf]

Supplemental Information 1	Definition of diseases
Supplemental Information 2	Supplemental figure 1. Measurement and identification of PLAs
Supplemental Information 3	Supplemental table 1. Univariate logistic regression for AKI
Supplemental Information 4	Supplemental figure 2. Estimated marginal means of indexes
Supplemental Information 5	Supplemental table 2. Odds ratios of PLAs
Supplemental Information 6	Supplemental table 3. Preoperative parameters and PLAs
Supplemental Information 7	Supplemental figure 3. ROC curve of baseline PLAs
Supplemental Information 8	Supplemental table 4. Multivariate logistic regression for AKI
Supplemental Information 9	Supplemental References

Supplemental Information 1: Definition of diseases

Acute respiratory distress syndrome (ARDS) was defined according to the Berlin definition: mild (200 mm Hg$<PaO_2/FIO_2\leq$300 mm Hg), moderate (100 mm$<$Hg$PaO_2/FIO_2\leq$200 mm Hg), and severe ($PaO_2/FIO_2\leq$100 mm Hg).(*1*)

Heart failure was defined as a complex clinical syndrome resulting from any structural or functional cardiac disorder that impaired the ability of the ventricle to fill with, or eject, blood.(*2*)

Chronic obstructive pulmonary disease (COPD) was defined as symptoms including chronic and progressive dyspnea, cough, and sputum production, characterized by airflow limitation ($FEV_1/FVC < 0.70$, $FEV_1 >$ 80% predicted).(*3*)

Pulmonary hypertension was defined as an increase in mean pulmonary arterial pressure (PAP) ≥ 25 mmHg at rest as assessed by right heart catheterization.(*4*)

A **pneumonia** was defined as a patient with chest radiograph with lobar or segmental consolidation, interstitial change, with or without pleural effusion and at least one of the following four signs, symptoms, or laboratory tests: 1) Recent cough, sticky sputum, or aggravating existent respiratory symptoms and purulent sputum, with or without pleural pain; 2) Fever; 3) Consolidation from physical examination and/or moist rales; 4) WBC>10x109/L or

WBC<4x109/L, increase in WBC early stage.(*5*)

Systemic inflammatory response syndrome (SIRS) was diagnosed when at least two SIRS criteria were fulfilled, including: 1) temperature >38°C or <36°C; 2) heart rate >90 bpm; 3) respiratory rate >20 breaths per minute, or arterial carbon dioxide tension <32 mm Hg (equivalent to 4.3 kPa); 4) white cell count >11 or <4 ($\times 10^9$ cells), or 10% immature (band) forms.(*6*)

Supplemental Information 2

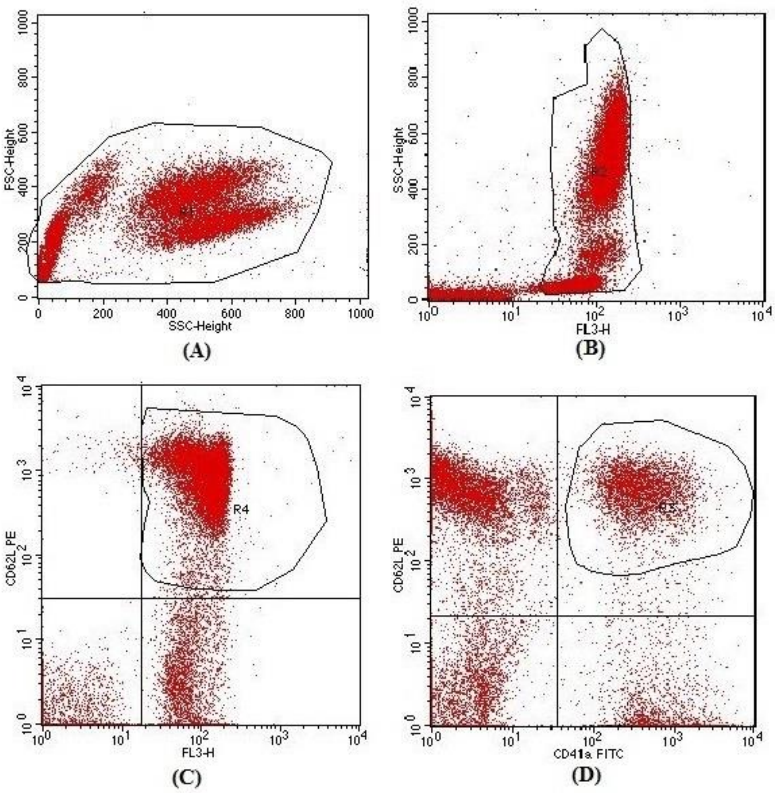

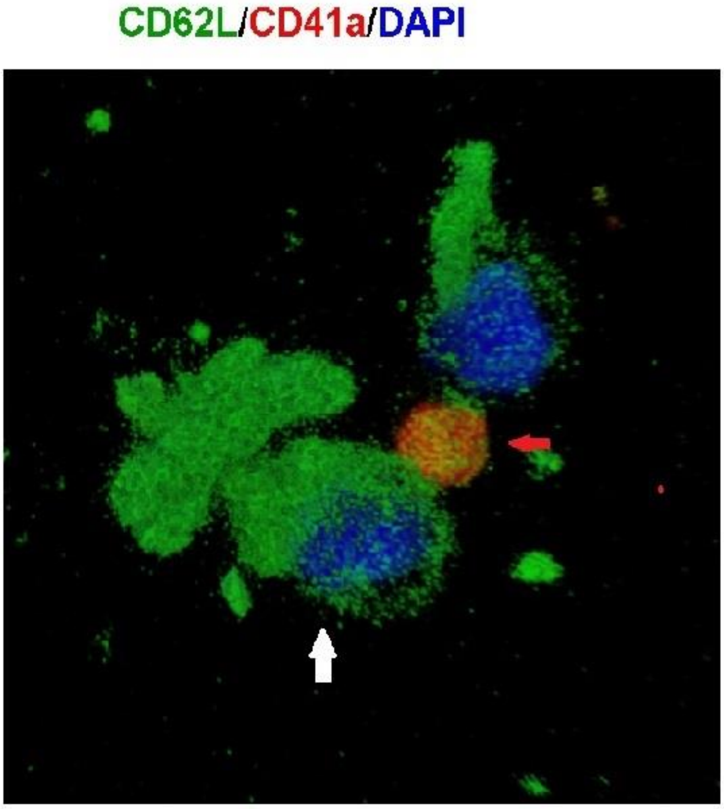

(E)

Supplemental figure 1. Measurement and identification of PLAs with flow cytometry (A-D) and

confocal fluorescence microscope (E). (A) Leukocytes were gated in forward scatter/side scatter dot plots. (B - C) Leukocytes were gated using FSC/SSC/FL3/CD62L. (D) PLAs were gated using CD62L/CD41a dot plots. (E) The samples were stained by FACSAria and observed under a confocal fluorescence microscope. The red arrow points to platelets, while the white arrow points to a leukocyte.

Supplemental Information 3

Supplemental table 1. Univariate logistic regression for AKI

	p	OR	95% Confident Interval	
			lower	upper
Age	0.59	1.02	0.96	1.08
Gender	**0.08**	0.39	0.13	1.10
Body mass index	0.79	1.02	0.85	1.23
Smoking	**0.02**	3.58	1.23	10.38
NYHA class	0.36	2.61	0.33	20.48
Diabetes	0.23	4.02	0.42	38.38
Atrial fibrillation	0.51	0.70	0.24	2.02
Hypertension	**0.07**	3.57	0.91	14.02
Left atrial thrombus	0.98	0.98	0.21	4.57
Medications				
Warfarin	0.10	8.11	0.69	94.93
Calcium antagonists	1.00	0.00	/	/
β blocker	0.42	1.92	0.40	9.21
Digoxin	0.61	0.58	0.07	4.62
Aspirin	1.00	0.00	/	/
Anticoagulants	0.87	1.19	0.14	9.74
Insulin	1.00	0.00	/	/
ACEI	1.00	0.00	/	/
Diuretics	0.61	0.58	0.07	4.62
baseline level				
Leukocytes	0.96	1.01	0.73	1.39
Platelets	0.13	0.99	0.98	1.00
Erythrocytes	0.43	1.47	0.57	3.78
TNF-α	0.36	1.06	0.94	1.19
IL-8	0.12	1.21	0.95	1.53
LVEF	0.89	1.00	0.95	1.07
EF SCORE	0.64	0.61	0.08	4.82
Type of valvular disease	0.16	1.64	0.82	3.29
Mitral and Aortic valve	reference			
Mitral valve	0.35	0.51	0.12	2.10
Aortic valve	0.95	2.77	0.84	9.16

CPB time	0.20	1.01	1.00	1.02
Cross-clamp time	0.65	1.00	0.99	1.02
Transfusion				
Red blood cell	0.62	0.89	0.57	1.40
Platelets	1.00	0.00	/	/
Fresh frozen plasma	0.83	1.00	0.99	1.00

Supplemental table 1. In bold, p<0.1; NYHA class, New York Heart Association functional class; ACEI, Angiotensin - converting enzyme inhibitor; LVEF, left ventricle ejection fraction; EF SCORE, European System for Cardiac Operative Risk Evaluation; CPB, cardiopulmonary bypass.

Supplemental Information 4

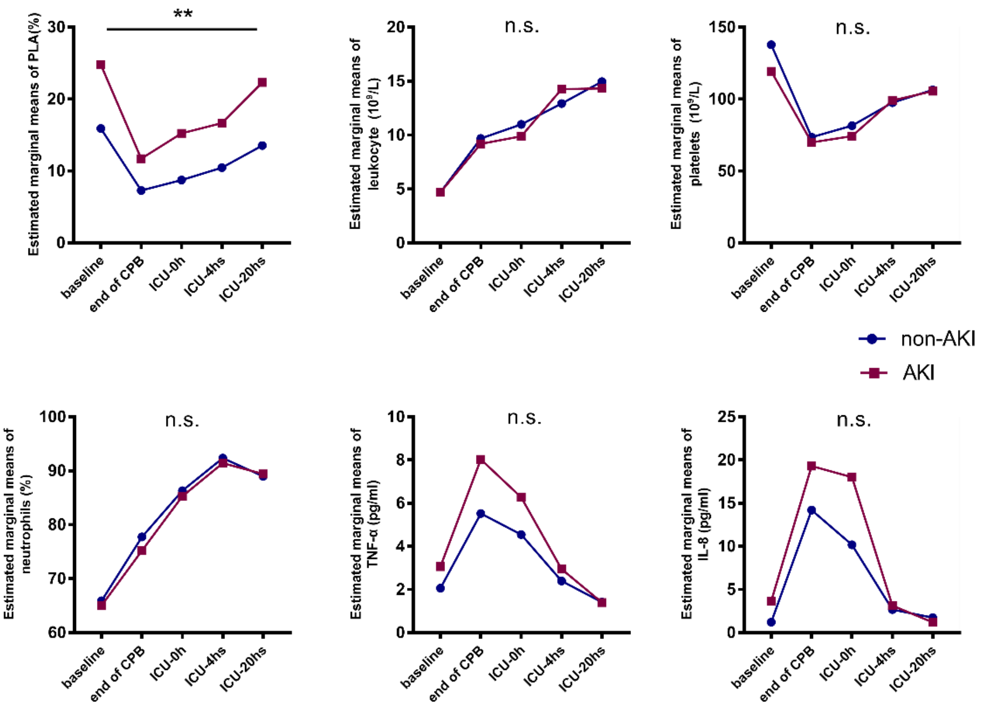

Supplemental figure 2. Estimated marginal means of indexes (analyzed by two-way repeated measure ANOVA). No significant difference was observed between AKI and non-AKI groups in leukocytes, neutrophils, platelets, TNF-α and IL-8 (p>0.05). PLAs in AKI group was significantly increased (p<0.01) during our observation.

Supplemental Information 5

Supplemental table 2. Odds ratios of PLAs

	Unadjusted	Adjusted#

	OR（95%CI）	p	OR（95%CI）	p
baseline	1.04（1.00-1.07）	0.03	1.05（1.01-1.09）	0.01
end of CPB	1.06（1.00-1.12）	0.04	1.07（1.01-1.14）	0.03
ICU-0h	1.07（1.02-1.13）	0.01	1.09（1.03-1.15）	0.00
ICU-4hs	1.05（1.01-1.10）	0.02	1.07（1.01-1.12）	0.01
ICU-20hs	1.05（1.01-1.10）	0.01	1.06（1.02-1.11）	0.01

Supplemental table 2. Odds ratios of PLAs at different time points. #Adjusted by gender, age, BMI, smoking history, NYHA classification, hypertension, diabetes, atrial fibrillation, digoxin, type of valvular disease and CPB time.

Supplemental Information 6

Supplemental table 3. Preoperative parameters and PLAs

	p	Odds ratio	95%CI	
Age	0.79	1.00	0.98	1.03
Gender	0.64	0.87	0.50	1.52
Body mass index	0.67	1.02	0.93	1.12
Smoking	0.64	1.16	0.62	2.19
NYHA class	0.87	1.06	0.52	2.16
Diabetes	0.39	2.64	0.29	23.97
Atrial fibrillation	0.25	1.36	0.81	2.27
Hypertension	0.65	0.80	0.30	2.10
Left atrial thrombus	0.26	1.58	0.72	3.49
Medications				
Warfarin	0.83	1.30	0.12	14.55
Calcium antagonists	1.00	/	/	/
β blocker	0.47	1.44	0.53	3.94
Digoxin	0.45	0.73	0.32	1.66
Aspirin	0.84	1.14	0.33	4.01
Anticoagulants	0.77	1.18	0.38	3.63
Insulin	1.00	/	/	/
ACEI	0.75	0.80	0.21	3.07
Diuretics	0.74	0.87	0.38	1.99
Baseline level				
Leukocytes	0.69	1.03	0.88	1.22

Platelets	0.11	1.00	1.00	1.01
Erythrocytes	0.08	1.58	0.94	2.64
TNF-α	0.29	0.96	0.88	1.04
IL-8	0.50	1.06	0.90	1.25
LVEF	0.73	1.01	0.98	1.04
EF SCORE	0.94	0.97	0.40	2.31

Supplemental table 3. Univariate logistic regression preoperative parameters and PLAs. PLAs was entered as categorical variable (cutoff value =6.8%, by ROC curve). No significant relationship has been found between these indexes and PLAs.

Supplemental Information 7

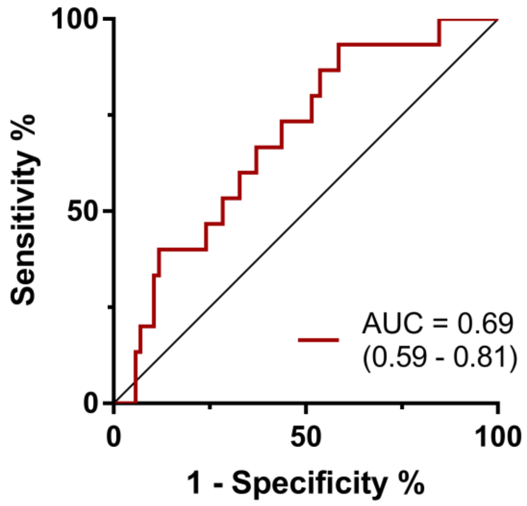

Supplemental figure 3. Receiver operating characteristic (ROC) curve analysis of baseline PLAs before surgery with a significant area under the curve (AUC) of 0.69 (95%CI 0.59 - 0.81) and sensitivity, specificity, positive predictive value and negative predictive value of 93.3%, 40.2%, 9.3% and 98.9%, respectively.

Supplemental Information 8

Supplemental table 4. Multivariate logistic regression for AKI

	B	S.E.	OR	95% CI		p
				lower	upper	
Age	0.00	0.03	1.00	0.94	1.07	0.94
Gender	0.00	1.15	1.00	0.10	9.55	1.00
Body mass index	-0.01	0.11	0.99	0.79	1.22	0.90

Smoking	0.94	1.18	2.57	0.26	25.71	0.42
NYHA class	-0.90	1.12	0.41	0.04	3.67	0.42
Diabetes	1.64	1.41	5.15	0.32	82.08	0.25
Atrial fibrillation	-0.23	0.71	0.79	0.20	3.21	0.74
Hypertension	0.63	0.89	1.88	0.33	10.76	0.48
Digoxin	-0.38	1.10	0.68	0.08	5.96	0.73
Type of valvular disease						
Mitral and Aortic valve	reference					0.51
Mitral valve	-0.20	0.81	0.82	0.17	4.04	0.81
Aortic valve	0.82	0.83	2.26	0.45	11.52	0.32
CPB time	0.01	0.01	1.01	0.99	1.03	0.18
Baseline level						
PLAs	0.05	0.02	1.05	1.01	1.09	**0.01**
Leukocytes	0.04	0.17	1.04	0.74	1.47	0.80
Platelets	-0.01	0.01	0.99	0.98	1.01	0.25
Neutrophils	-0.02	0.03	0.98	0.92	1.05	0.58
TNF-α	0.09	0.07	1.10	0.96	1.26	0.19
IL-8	0.19	0.11	1.21	0.98	1.50	0.07

Supplemental table 4. In bold, $p<0.05$. The baseline of PLAs was an independent risk factor for postoperative AKI.

Supplemental Information 9

Supplemental References

1. A. D. T. Force *et al.*, Acute respiratory distress syndrome: the Berlin Definition. *JAMA* **307**, 2526-2533 (2012).
2. S. A. Hunt *et al.*, ACC/AHA Guidelines for the Evaluation and Management of Chronic Heart Failure in the Adult: Executive Summary A Report of the American College of Cardiology/American Heart Association Task Force on Practice Guidelines (Committee to Revise the 1995 Guidelines for the Evaluation and Management of Heart Failure): Developed in Collaboration With the International Society for Heart and Lung Transplantation; Endorsed by the Heart Failure Society of America. *Circulation* **104**, 2996-3007 (2001).
3. K. F. Rabe *et al.*, Global strategy for the diagnosis, management, and prevention of chronic obstructive pulmonary disease: GOLD executive summary. *Am J Respir Crit Care Med* **176**, 532-555 (2007).
4. N. Galie *et al.*, Guidelines for the diagnosis and treatment of pulmonary hypertension: the Task Force for the Diagnosis and Treatment of Pulmonary Hypertension of the European Society of Cardiology (ESC) and the European Respiratory Society (ERS), endorsed by the International Society of Heart and Lung Transplantation (ISHLT). *Eur Heart J* **30**, 2493-2537 (2009).

5. L. X. He, Guidelines for the diagnosis and treatment of community-acquired pneumonia: learning and practicing. *Zhonghua Jie He He Hu Xi Za Zhi* **29**, 649-650 (2006).
6. R. C. Bone *et al.*, Definitions for sepsis and organ failure and guidelines for the use of innovative therapies in sepsis. The ACCP/SCCM Consensus Conference Committee. American College of Chest Physicians/Society of Critical Care Medicine. *Chest* **101**, 1644-1655 (1992).
